# *catena*-Poly[[bis­[2-(2-pyrid­yl)-1-*H*-imidazole-κ^2^ *N* ^2^,*N* ^3^]cadmium]-μ-benzene-1,3-dicarboxyl­ato-κ^2^ *O* ^1^:*O* ^3^]

**DOI:** 10.1107/S1600536811001243

**Published:** 2011-01-29

**Authors:** Chun-Jiang Li, Jing-Mei Lu, Fan Tu, Jing-Ying Chen, Yu-Jia Li

**Affiliations:** a Faculty of Life Sciences, Northeast Normal University, Changchun 130024, People’s Republic of China; b School of Basic Medical Sciences, Jiamusi University, Jiamusi 154007, People’s Republic of China

## Abstract

In the title coordinaltion polymer, [Cd(C_8_H_4_O_4_)(C_8_H_7_N_3_)_2_]_*n*_, the Cd^II^ atom, lying on a twofold rotation axis, is six-coordinated by two carboxyl­ate O atoms from two benzene-1,3-dicarboxyl­ate (*m*-BDC) ligands and four N atoms from two chelating 2-(2-pyrid­yl)imidazole mol­ecules, forming a slightly distorted octa­hedral geometry. The *m*-BDC ligand is located over a twofold rotation axis. The Cd^II^ atoms are bridged by the *m*-BDC ligands, leading to a wave-shaped chain structure along [010]. N—H⋯O hydrogen bonds connect the chains.

## Related literature

For general background to metal-organic frameworks, see: Chen *et al.* (2009 ▶); Rosi *et al.* (2003 ▶); Su *et al.* (2004 ▶); Xiao *et al.* (2006 ▶). For compounds based on benzene-1,3-dicarboxylate ligands, see: Banerjee *et al.* (2008 ▶); Che *et al.* (2009 ▶); Clegg & Russo (2009 ▶); Li *et al.* (2008 ▶); Su *et al.* (2009 ▶); Zhao (2008 ▶).
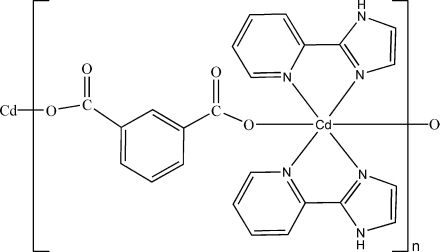


## Experimental

### 

#### Crystal data

[Cd(C_8_H_4_O_4_)(C_8_H_7_N_3_)_2_]
*M* *_r_* = 566.85
Orthorhombic, 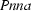

*a* = 8.720 (5) Å
*b* = 20.102 (4) Å
*c* = 13.483 (5) Å
*V* = 2363.4 (17) Å^3^
*Z* = 4
Mo *K*α radiation
μ = 0.97 mm^−1^
*T* = 293 K
0.10 × 0.08 × 0.06 mm

#### Data collection

Bruker APEX CCD diffractometer
Absorption correction: multi-scan (*SADABS*; Sheldrick, 1996 ▶) *T*_min_ = 0.911, *T*_max_ = 0.944
11996 measured reflections
2330 independent reflections
1298 reflections with *I* > 2σ(*I*)
*R*_int_ = 0.060

#### Refinement

*R*[*F* ^2^ > 2σ(*F* ^2^)] = 0.034
*wR*(*F* ^2^) = 0.100
*S* = 1.00
2330 reflections
164 parameters
75 restraints
H atoms treated by a mixture of independent and constrained refinement
Δρ_max_ = 0.39 e Å^−3^
Δρ_min_ = −0.69 e Å^−3^

### 

Data collection: *SMART* (Bruker, 2007 ▶); cell refinement: *SAINT* (Bruker, 2007 ▶); data reduction: *SAINT*; program(s) used to solve structure: *SHELXS97* (Sheldrick, 2008 ▶); program(s) used to refine structure: *SHELXL97* (Sheldrick, 2008 ▶); molecular graphics: *SHELXTL* (Sheldrick, 2008 ▶) and *DIAMOND* (Brandenburg, 1999 ▶); software used to prepare material for publication: *SHELXTL*.

## Supplementary Material

Crystal structure: contains datablocks I, global. DOI: 10.1107/S1600536811001243/hy2397sup1.cif

Structure factors: contains datablocks I. DOI: 10.1107/S1600536811001243/hy2397Isup2.hkl

Additional supplementary materials:  crystallographic information; 3D view; checkCIF report

## Figures and Tables

**Table 1:** Selected bond lengths (Å)

Cd1—O1	2.257 (3)
Cd1—N1	2.288 (4)
Cd1—N3	2.461 (4)

**Table 2:** Hydrogen-bond geometry (Å, °)

*D*—H⋯*A*	*D*—H	H⋯*A*	*D*⋯*A*	*D*—H⋯*A*
N2—H1*N*⋯O2^i^	0.95 (5)	1.80 (5)	2.741 (5)	166 (5)

Symmetry code: (i) 
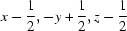
.

**Fig. 1.**
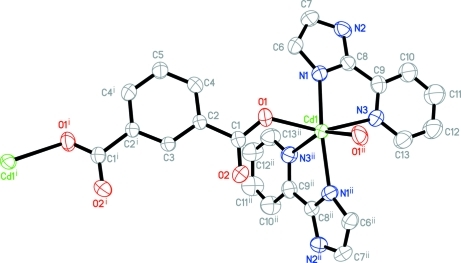
The structure of the title compound. Displacement ellipsoids are drawn at the 30% probability level. H atoms have been omitted for clarity. [Symmetry codes: (i) -x+5/2, -y+1, z; (ii) x, -y+1/2, -z+3/2.]

**Fig. 2.**
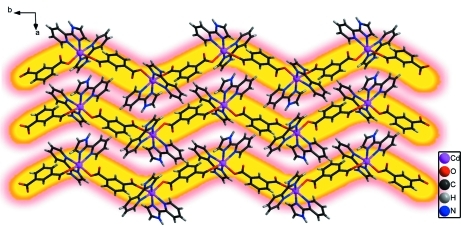
View of the one-dimensional chain structure of the title compound.

## supplementary crystallographic information

### Comment

Currently, the design and synthesis of metal-organic frameworks (MOFs) are of great interest owing to their intriguing variety of architectures and their tremendous potential applications in many fields (Chen *et al.*, 2009; Rosi *et al.*, 2003; Su *et al.*, 2004; Xiao *et al.*, 2006). As a multidentate ligand, benzene-1,3-dicarboxylic acid (*m*-H_2_BDC) has two carboxyl groups and, therefore, has been widely reported as a good candidate not only in the construction of various coordination polymers but also in the construction of MOFs with multi-dimension (Banerjee *et al.*, 2008; Che *et al.*, 2009; Clegg & Russo, 2009; Li *et al.*, 2008; Su *et al.*, 2009; Zhao, 2008).

In the title coordinaltion polymer (Fig. 1), the central Cd^II^ ion is six-coordinated by two carboxylate O atoms from two *m*-BDC ligands and four N atoms from two 2-(2-pyridyl)imidazole (2-PyIM) molecules (Table 1), forming a slightly distorted octahedral geometry. The Cd^II^ ions are bridged by the *m*-BDC ligands, leading to a wave-shaped chain structure, as shown in Fig. 2. N—H···O hydrogen bonds connect the chains (Table 2).

### Experimental

All chemicals were purchased from commercial sources and used without further purification. CdSO_4_.8H_2_O (0.033 mmol), *m*-H_2_BDC (0.1 mmol) and 2-PyIM (0.2 mmol) were dissolved in 15 ml of water. The mixture was stirred for 2 h at room temperature and then heated in a 30 ml Teflon-lined stainless steel autoclave for 3 d at 323 K. After the autoclave was cooled to room temperature, colorless block crystals were harvested.

### Refinement

C-bound H atoms were positioned geometrically and refined as riding atoms, with C—H = 0.93 Å and *U*_iso_(H) = 1.2*U*_eq_(C). The H atom on imidazole N2 was located from a difference Fourier map and refined isotropically.

### Crystal data

**Table:**

[Cd(C_8_H_4_O_4_)(C_8_H_7_N_3_)_2_]	*F*(000) = 1136
*M**_r_* = 566.85	*D*_x_ = 1.593 Mg m^−^^3^*D*_m_ = 1.593 Mg m^−^^3^*D*_m_ measured by not measured
Orthorhombic, *P**n**n**a*	Mo *K*α radiation, λ = 0.71073 Å
Hall symbol: -P 2a 2bc	Cell parameters from 2329 reflections
*a* = 8.720 (5) Å	θ = 2.0–52.0°
*b* = 20.102 (4) Å	µ = 0.97 mm^−^^1^
*c* = 13.483 (5) Å	*T* = 293 K
*V* = 2363.4 (17) Å^3^	Block, colorless
*Z* = 4	0.10 × 0.08 × 0.06 mm

### Data collection

**Table:**

Bruker APEX CCD diffractometer	2330 independent reflections
Radiation source: fine-focus sealed tube	1298 reflections with *I* > 2σ(*I*)
graphite	*R*_int_ = 0.060
φ and ω scans	θ_max_ = 26.0°, θ_min_ = 1.8°
Absorption correction: multi-scan (*SADABS*; Sheldrick, 1996)	*h* = −10→10
*T*_min_ = 0.911, *T*_max_ = 0.944	*k* = −21→24
11996 measured reflections	*l* = −16→16

### Refinement

**Table:**

Refinement on *F*^2^	Primary atom site location: structure-invariant direct methods
Least-squares matrix: full	Secondary atom site location: difference Fourier map
*R*[*F*^2^ > 2σ(*F*^2^)] = 0.034	Hydrogen site location: inferred from neighbouring sites
*wR*(*F*^2^) = 0.100	H atoms treated by a mixture of independent and constrained refinement
*S* = 1.00	*w* = 1/[σ^2^(*F*_o_^2^) + (0.051*P*)^2^] where *P* = (*F*_o_^2^ + 2*F*_c_^2^)/3
2330 reflections	(Δ/σ)_max_ < 0.001
164 parameters	Δρ_max_ = 0.39 e Å^−^^3^
75 restraints	Δρ_min_ = −0.69 e Å^−^^3^

### Fractional atomic coordinates and isotropic or equivalent isotropic displacement parameters (Å^2^)

**Table:**

	*x*	*y*	*z*	*U*_iso_*/*U*_eq_	
Cd1	0.96428 (5)	0.2500	0.7500	0.05598 (19)	
O1	1.1284 (4)	0.33138 (14)	0.7049 (2)	0.0738 (8)	
C2	1.2020 (5)	0.44358 (18)	0.6952 (3)	0.0589 (10)	
O2	1.1291 (4)	0.39000 (15)	0.8440 (2)	0.0883 (10)	
C9	0.7428 (5)	0.1504 (2)	0.6230 (4)	0.0691 (12)	
C3	1.2500	0.5000	0.7444 (4)	0.0549 (13)	
H3	1.2500	0.5000	0.8133	0.066*	
N3	0.7725 (5)	0.16257 (19)	0.7191 (3)	0.0692 (10)	
N2	0.7945 (5)	0.1991 (2)	0.4546 (3)	0.0759 (12)	
C13	0.7096 (7)	0.1223 (3)	0.7857 (4)	0.0919 (16)	
H13	0.7293	0.1300	0.8525	0.110*	
N1	0.9098 (5)	0.24430 (17)	0.5842 (3)	0.0673 (10)	
C8	0.8139 (5)	0.1966 (2)	0.5556 (3)	0.0621 (11)	
C6	0.9516 (6)	0.2781 (3)	0.4998 (4)	0.0769 (15)	
H6	1.0177	0.3144	0.4980	0.092*	
C1	1.1489 (6)	0.3835 (2)	0.7533 (3)	0.0630 (9)	
C4	1.2024 (6)	0.4443 (2)	0.5936 (3)	0.0934 (15)	
H4	1.1702	0.4070	0.5587	0.112*	
C10	0.6521 (6)	0.0978 (3)	0.5917 (4)	0.0947 (16)	
H10	0.6344	0.0903	0.5246	0.114*	
C11	0.5901 (6)	0.0577 (3)	0.6615 (5)	0.1110 (17)	
H11	0.5292	0.0218	0.6429	0.133*	
C12	0.6178 (8)	0.0702 (3)	0.7606 (4)	0.1049 (17)	
H12	0.5744	0.0435	0.8094	0.126*	
C7	0.8821 (8)	0.2507 (3)	0.4193 (4)	0.0852 (15)	
H7	0.8919	0.2642	0.3537	0.102*	
C5	1.2500	0.5000	0.5431 (5)	0.158 (5)	
H5	1.2500	0.5000	0.4741	0.189*	
H1N	0.735 (6)	0.174 (3)	0.409 (4)	0.14 (2)*	

### Atomic displacement parameters (Å^2^)

**Table:**

	*U*^11^	*U*^22^	*U*^33^	*U*^12^	*U*^13^	*U*^23^
Cd1	0.0763 (3)	0.0434 (3)	0.0482 (3)	0.000	0.000	0.0039 (2)
O1	0.0959 (19)	0.0469 (15)	0.0786 (16)	−0.0127 (15)	0.0124 (16)	−0.0040 (14)
C2	0.086 (3)	0.041 (2)	0.0496 (19)	−0.006 (2)	0.000 (2)	−0.0011 (18)
O2	0.139 (3)	0.064 (2)	0.061 (2)	−0.016 (2)	0.000 (2)	0.0127 (16)
C9	0.062 (3)	0.063 (3)	0.083 (3)	0.002 (2)	0.001 (3)	−0.001 (3)
C3	0.075 (3)	0.046 (3)	0.045 (3)	0.000 (3)	0.000	0.000
N3	0.076 (3)	0.066 (2)	0.066 (2)	−0.011 (2)	0.007 (2)	0.0065 (19)
N2	0.094 (3)	0.072 (3)	0.062 (3)	0.005 (2)	−0.018 (2)	−0.010 (2)
C13	0.101 (4)	0.100 (4)	0.075 (3)	−0.020 (4)	0.006 (3)	0.007 (3)
N1	0.088 (3)	0.060 (2)	0.054 (2)	−0.007 (2)	−0.008 (2)	0.0039 (19)
C8	0.074 (3)	0.056 (3)	0.057 (3)	0.006 (2)	−0.009 (2)	−0.002 (2)
C6	0.106 (4)	0.061 (3)	0.064 (3)	−0.011 (3)	−0.004 (3)	0.012 (3)
C1	0.083 (2)	0.0461 (18)	0.0602 (19)	−0.0035 (17)	0.000 (2)	−0.0019 (19)
C4	0.152 (4)	0.071 (3)	0.057 (2)	−0.041 (3)	0.001 (3)	−0.010 (2)
C10	0.090 (4)	0.099 (4)	0.095 (4)	−0.028 (3)	0.000 (3)	−0.003 (3)
C11	0.100 (4)	0.125 (4)	0.108 (3)	−0.033 (3)	0.004 (3)	−0.001 (3)
C12	0.105 (4)	0.114 (4)	0.096 (3)	−0.033 (4)	0.005 (3)	0.009 (3)
C7	0.121 (4)	0.082 (4)	0.052 (3)	0.004 (4)	−0.007 (3)	0.014 (3)
C5	0.319 (13)	0.108 (6)	0.046 (4)	−0.116 (8)	0.000	0.000

### Geometric parameters (Å, °)

**Table:**

Cd1—O1	2.257 (3)	C13—C12	1.360 (8)
Cd1—N1	2.288 (4)	C13—H13	0.9300
Cd1—N3	2.461 (4)	N1—C8	1.330 (5)
O1—C1	1.246 (5)	N1—C6	1.376 (6)
C2—C4	1.370 (5)	C6—C7	1.359 (7)
C2—C3	1.378 (4)	C6—H6	0.9300
C2—C1	1.512 (6)	C4—C5	1.374 (5)
O2—C1	1.243 (4)	C4—H4	0.9300
C9—N3	1.343 (5)	C10—C11	1.352 (7)
C9—C10	1.388 (6)	C10—H10	0.9300
C9—C8	1.439 (6)	C11—C12	1.380 (6)
C3—C2^i^	1.378 (4)	C11—H11	0.9300
C3—H3	0.9300	C12—H12	0.9300
N3—C13	1.328 (6)	C7—H7	0.9300
N2—C7	1.373 (6)	C5—C4^i^	1.374 (5)
N2—C8	1.374 (5)	C5—H5	0.9300
N2—H1N	0.95 (5)		
			
O1^ii^—Cd1—O1	101.29 (17)	N3—C13—H13	118.5
O1^ii^—Cd1—N1^ii^	84.54 (13)	C12—C13—H13	118.5
O1—Cd1—N1^ii^	111.01 (12)	C8—N1—C6	106.5 (4)
O1^ii^—Cd1—N1	111.01 (12)	C8—N1—Cd1	116.8 (3)
O1—Cd1—N1	84.54 (13)	C6—N1—Cd1	136.8 (3)
N1^ii^—Cd1—N1	156.0 (2)	N1—C8—N2	109.7 (4)
O1^ii^—Cd1—N3	87.64 (13)	N1—C8—C9	123.5 (4)
O1—Cd1—N3	154.52 (13)	N2—C8—C9	126.7 (5)
N1^ii^—Cd1—N3	93.45 (13)	C7—C6—N1	110.0 (4)
N1—Cd1—N3	69.99 (13)	C7—C6—H6	125.0
O1^ii^—Cd1—N3^ii^	154.52 (13)	N1—C6—H6	125.0
O1—Cd1—N3^ii^	87.64 (13)	O2—C1—O1	125.7 (4)
N1^ii^—Cd1—N3^ii^	69.99 (13)	O2—C1—C2	117.9 (4)
N1—Cd1—N3^ii^	93.45 (13)	O1—C1—C2	116.4 (4)
N3—Cd1—N3^ii^	94.41 (19)	C2—C4—C5	120.4 (5)
C1—O1—Cd1	124.0 (3)	C2—C4—H4	119.8
C4—C2—C3	118.1 (4)	C5—C4—H4	119.8
C4—C2—C1	121.8 (4)	C11—C10—C9	118.1 (5)
C3—C2—C1	120.1 (4)	C11—C10—H10	121.0
N3—C9—C10	122.9 (4)	C9—C10—H10	121.0
N3—C9—C8	114.1 (4)	C10—C11—C12	119.7 (6)
C10—C9—C8	123.0 (5)	C10—C11—H11	120.2
C2—C3—C2^i^	122.6 (5)	C12—C11—H11	120.2
C2—C3—H3	118.7	C13—C12—C11	119.0 (5)
C2^i^—C3—H3	118.7	C13—C12—H12	120.5
C13—N3—C9	117.5 (4)	C11—C12—H12	120.5
C13—N3—Cd1	127.0 (4)	C6—C7—N2	106.2 (4)
C9—N3—Cd1	115.1 (3)	C6—C7—H7	126.9
C7—N2—C8	107.7 (4)	N2—C7—H7	126.9
C7—N2—H1N	119 (3)	C4^i^—C5—C4	120.5 (6)
C8—N2—H1N	133 (3)	C4^i^—C5—H5	119.7
N3—C13—C12	123.0 (5)	C4—C5—H5	119.7
			
O1^ii^—Cd1—O1—C1	−109.2 (4)	N3—Cd1—N1—C6	177.0 (5)
N1^ii^—Cd1—O1—C1	−20.8 (4)	N3^ii^—Cd1—N1—C6	83.6 (5)
N1—Cd1—O1—C1	140.4 (4)	C6—N1—C8—N2	0.4 (5)
N3—Cd1—O1—C1	142.1 (4)	Cd1—N1—C8—N2	−179.2 (3)
N3^ii^—Cd1—O1—C1	46.7 (4)	C6—N1—C8—C9	−179.4 (4)
C4—C2—C3—C2^i^	−0.1 (3)	Cd1—N1—C8—C9	1.1 (6)
C1—C2—C3—C2^i^	−179.3 (4)	C7—N2—C8—N1	−0.1 (6)
C10—C9—N3—C13	−0.8 (7)	C7—N2—C8—C9	179.6 (4)
C8—C9—N3—C13	179.5 (4)	N3—C9—C8—N1	4.8 (6)
C10—C9—N3—Cd1	172.1 (4)	C10—C9—C8—N1	−175.0 (4)
C8—C9—N3—Cd1	−7.6 (5)	N3—C9—C8—N2	−174.9 (4)
O1^ii^—Cd1—N3—C13	64.9 (4)	C10—C9—C8—N2	5.3 (7)
O1—Cd1—N3—C13	176.6 (4)	C8—N1—C6—C7	−0.5 (6)
N1^ii^—Cd1—N3—C13	−19.4 (4)	Cd1—N1—C6—C7	178.9 (4)
N1—Cd1—N3—C13	178.3 (4)	Cd1—O1—C1—O2	26.3 (7)
N3^ii^—Cd1—N3—C13	−89.6 (4)	Cd1—O1—C1—C2	−153.4 (3)
O1^ii^—Cd1—N3—C9	−107.2 (3)	C4—C2—C1—O2	−168.9 (5)
O1—Cd1—N3—C9	4.5 (5)	C3—C2—C1—O2	10.2 (6)
N1^ii^—Cd1—N3—C9	168.4 (3)	C4—C2—C1—O1	10.9 (7)
N1—Cd1—N3—C9	6.2 (3)	C3—C2—C1—O1	−170.0 (4)
N3^ii^—Cd1—N3—C9	98.2 (3)	C3—C2—C4—C5	0.3 (7)
C9—N3—C13—C12	−0.1 (8)	C1—C2—C4—C5	179.4 (4)
Cd1—N3—C13—C12	−172.1 (5)	N3—C9—C10—C11	0.7 (8)
O1^ii^—Cd1—N1—C8	75.6 (3)	C8—C9—C10—C11	−179.6 (5)
O1—Cd1—N1—C8	175.6 (3)	C9—C10—C11—C12	0.3 (9)
N1^ii^—Cd1—N1—C8	−52.2 (3)	N3—C13—C12—C11	1.1 (10)
N3—Cd1—N1—C8	−3.6 (3)	C10—C11—C12—C13	−1.2 (9)
N3^ii^—Cd1—N1—C8	−97.1 (3)	N1—C6—C7—N2	0.4 (6)
O1^ii^—Cd1—N1—C6	−103.8 (5)	C8—N2—C7—C6	−0.2 (6)
O1—Cd1—N1—C6	−3.7 (5)	C2—C4—C5—C4^i^	−0.1 (3)
N1^ii^—Cd1—N1—C6	128.4 (5)		

Symmetry codes: (i) −*x*+5/2, −*y*+1, *z*; (ii) *x*, −*y*+1/2, −*z*+3/2.

### Hydrogen-bond geometry (Å, °)

**Table:**

*D*—H···*A*	*D*—H	H···*A*	*D*···*A*	*D*—H···*A*
N2—H1N···O2^iii^	0.95 (5)	1.80 (5)	2.741 (5)	166 (5)

Symmetry codes: (iii) *x*−1/2, −*y*+1/2, *z*−1/2.
